# *Cordyceps militaris* induces apoptosis in ovarian cancer cells through TNF-α/TNFR1-mediated inhibition of NF-κB phosphorylation

**DOI:** 10.1186/s12906-019-2780-5

**Published:** 2020-01-13

**Authors:** Eunbi Jo, Hyun-Jin Jang, Kyeong Eun Yang, Min Su Jang, Yang Hoon Huh, Hwa-Seung Yoo, Jun Soo Park, Ik-Soon Jang, Soo Jung Park

**Affiliations:** 1 Division of Analytical Science, Korea Basic Science Institute, Gwahangno 113, Yuseong-gu, Daejeon, 305-333 Republic of Korea; 2 Department of Biological Sciences, Sungkyunkwan University, Suwon, 16419 Republic of Korea; 3 Division of Biological Science and Technology, Yonsei University, Wonju, 220-100 Republic of Korea; 4 Electron Microscopy Research Center, Korea Basic Science Institute, Cheongju, 28119 Republic of Korea; 5 East-West Cancer Center, Daejeon University, Daejeon, 302-120 South Korea; 6 Division of Analytical Science, University of Science and Technology, Daejeon, 34113 Republic of Korea; 7 Department of Sasang Constitutional Medicine, College of Korean Medicine, Woosuk University, Wanju, Jeonbuk 55338 Republic of Korea

**Keywords:** Apoptosis, TNF-α, TNFR1, NF-κB, *C. militaris*

## Abstract

**Background:**

*Cordyceps militaris* (L.) Fr. (*C. militaris*) exhibits pharmacological activities, including antitumor properties, through the regulation of the nuclear factor kappa B (NF-κB) signaling. Tumor Necrosis Factor (TNF) and TNF-α modulates cell survival and apoptosis through NF- κB signaling. However, the mechanism underlying its mode of action on the NF-κB pathway is unclear.

**Methods:**

Here, we analyzed the effect of *C. militaris* extract (CME) on the proliferation of ovarian cancer cells by confirming viability, morphological changes, migration assay. Additionally, CME induced apoptosis was determined by apoptosis assay and apoptotic body formation under TEM. The mechanisms of CME were determined through microarray, immunoblotting and immunocytochemistry.

**Results:**

CME reduced the viability of cells in a dose-dependent manner and induced morphological changes. We confirmed the decrease in the migration activity of SKOV-3 cells after treatment with CME and the consequent induction of apoptosis. Immunoblotting results showed that the CME-mediated upregulation of tumor necrosis factor receptor 1 (TNFR1) expression induced apoptosis of SKOV-3 cells via the serial activation of caspases. Moreover, CME negatively modulated NF-κB activation via TNFR expression, suggestive of the activation of the extrinsic apoptotic pathway. The binding of TNF-α to TNFR results in the disassociation of IκB from NF-κB and the subsequent translocation of the active NF-κB to the nucleus. CME clearly suppressed NF-κB translocation induced by interleukin (IL-1β) from the cytosol into the nucleus. The decrease in the expression levels of B cell lymphoma (Bcl)-xL and Bcl-2 led to a marked increase in cell apoptosis.

**Conclusion:**

These results suggest that *C. militaris* inhibited ovarian cancer cell proliferation, survival, and migration, possibly through the coordination between TNF-α/TNFR1 signaling and NF-κB activation. Taken together, our findings provide a new insight into a novel treatment strategy for ovarian cancer using *C. militaris*.

## Background

Epithelial ovarian cancer (EOC) is a histopathologically, morphologically, and molecularly heterogeneous group of neoplasms [1], and leads cause of gynecological malignancy-related deaths in women, with ~ 152,000 deaths worldwide yearly [2]. Standard Treatment for epithelial ovarian cancers (EOCs) is based on a combination of surgery and chemotherapy. The carboplatin/paclitaxel doublet remains the chemotherapy backbone for the initial treatment of ovarian cancer [3]. However, Resistance against chemotherapeutic agents often develops in ovarian cancer patients, contributing to high recurrence rates. In addition, induction of multidrug resistant and incurable tumor recurrence in the majority of patients after initial good response to standard carboplatin/taxane-based treatment are also significant factors contributing to this deadly disease [4].

Herbal medicine (HM), also called botanical medicine, refers to herbal materials, that contain parts of plants or other materials as active ingredients [5]. The combination of traditional herbal medicine and chemotherapy drugs is a therapy method with Asia characteristics, especially China and Korea. The traditional herbal medicine can increase the sensitivity of chemotherapy and reduce its side effects [6]. However, the detailed molecular mechanism underlining this has not been fully elucidated.

*Cordyceps militaris* (L.) Fr. is a species of fungus in the family Clavicipitaceae that has been a traditional potential harbour of bio-metabolites for herbal drugs in Korea and China for revitalization of various systems of the body including enhance of longevity and vitality [7, 8]. It contains many kinds of active ingredients (such as cordycepin, cordycepic acid, sterols (ergosterol), nucleosides, and polysaccharides), and due to its various physiological activities, it is now used for multiple medicinal purposes [9]. Evidence showed that the active principles of *C. militaris* are beneficial to act as immunomodulatory, anti-inflammatory, antimicrobial, antitumor, and antioxidant although the primary pharmacological activity slightly varies depending on the main ingredients in its extract [10, 11]. Both in vivo and in vitro experiments have demonstrated the anti-proliferative and apoptotic activities of *C. militaris* extract (CME) against human tumor cell lines. CME was demonstrated antitumor effects mainly through other various researched that suggested the induction of cell death and apoptosis, inhibition of angiogenesis, and suppression of invasion and metastasis by CME in human cancer cells [12–15]. *Cordyceps militaris* has recently received considerable attention as a potential source of anticancer drugs [16]. We found that *C. militaris* reduced the viability and migration activities, indicative of its potential ability to mediate apoptosis. In addition, in our previous researches, we investigated the anticancer effect of cordycepin that is major compound in *C. militaris* on human lung, renal, and ovarian cancer cells [17–21]. However, the molecular mechanism underlying the inhibitory effects of *C. militaris* on tumor cell proliferation and metastasis remains unclear.

Tumor necrosis factor (TNF), known for its cytotoxic functions, is involved in the regulation of proliferation, differentiation, and apoptosis or inflammation in a variety of cell types via nuclear factor kappa B (NF-κB) signaling [22–24]. TNF-α acts as a ligand and exerts two major effects. First, TNF-α induces apoptosis through the regulation of the expression of related genes [25, 26] and results in the condensation of chromatin, degradation of DNA through the activation of endogenous nucleases, and dissolution of cell into small membrane-bound apoptotic vesicles [27, 28]. Second, TNF-α has also been shown to induce cell survival and proliferation through a variety of signaling pathways associated with development, homeostasis, and oncogenic transformation [29–31]. Thus, the two characteristic functions of TNF-α are attributed to the presence of various subtypes of TNF receptors (TNFRs). This heterogeneous response to TNF-α is mediated following its binding to specific cell surface receptors, resulting in the activation of different signaling pathways. There are two types of TNFRs, namely, type 1 (TNFR1, also known TNFRSF1A) and type 2 (TNFR2, also known TNFRSF2). TNF-α signaling occurs through TNFR1 and/or TNFR2, leading to the activation of multiple signal pathways, including NF-κB pathway [28].

TNFR1 is expressed in almost all cell types, except red blood cells, while TNFR2 is abundant not only on immune cells but also on endothelial and hematopoietic cells. TNF-α binds to both receptors with high affinity. Binding of TNFR1 and TNFR2 to TNF-α activates or inhibits NF-κB and c-Jun N-terminal kinase (JNK)/stress-activated protein kinase pathways, both of which mediate cell activation, gene transcription, and cell survival [32, 33]. In particular, TNFR2 signaling induces cell survival and proliferation via NF-κB activation, eventually promoting development of cancer. In other words, TNFR2 signaling results in the activation of anti-apoptosis pathway [34], whereas the death domain-containing TNFR1 triggers apoptosis following binding of TNF-α through the inhibition of NF-kB activation [35]. Based on the cellular context, conditions, and microenvironment, TNFR activation may lead to the induction of proliferation, apoptosis, or necroptosis. Activation of these different cellular responses reflects the existence of a complex regulatory network after receptor activation [36].

In this study, we attempted to evaluate the effects of CME to promote TNF-α/TNFR-mediated signaling and apoptosis induction in human ovarian cancer cells. The data reported herein clearly demonstrate the involvement of CME in TNF-α/TNFR signaling via the downregulation of NF-κB activation, and the consequent activation of caspase-mediated pathways. We show that *C. militaris* prevented NF-κB activation by upregulating the expression of TNFR1 and that the subsequent activation of the extrinsic apoptotic process resulted in the induction of cancer cell death. Therefore, it is expected that targeting TNF-α/TNFR signaling and downstream NF-κB molecules activation through CME should improve ovarian cancer cell sensitivity to platinum based chemotherapy.

## Methods

### Preparation of alcoholic *C. militaris* extract

The fungus strain *C. militaris* was obtained from Wonkwang University’s College of Medicine (Jeollabuk-do, Republic of Korea). Fresh bodies or mycelia of *C. militaris* were extracted with 50% ethanol (w.v) at 80 °C for 3 h (five times). The CME was filtered using 1-μm pore-size filters, concentrated, sterilized, and dried as described in our previous study [37]. Extracts (200 g, yield [w/w)], 11%) were diluted with PBS for in vitro experiments.

### Reagents and chemicals

Fetal bovine serum (FBS), antibiotic-antimycotic (100×), and phosphate-buffered saline (PBS) were procured from Gibco™ (Waltham, MA, USA). Dulbecco’s modified Eagle’s medium (DMEM) was purchased from PAN-Biotech GmbH (Am Gewerbepark 13, 94,501 Aidenbach, Germany). Muse Annexin V & Dead Cell reagent was obtained from Millipore. Whole cell lysis buffer was procured from iNtRON™ Biotechnology Inc. (Seoul, Korea). Antibodies against B cell lymphoma (Bcl)-xL (1:500; cat. no. #2764), Bcl-2 (1:500; cat. no. #15071), caspase-3 (1:500; cat. no. #9662), and caspase-9 (1:500; cat. no. #9502) were supplied by Cell Signaling (Beverly, MA, USA) and those against TNFR1 (1:200; cat. no. sc-8436) and β-actin (1:1000; cat. no. sc-47,778) were obtained from Santa Cruz (Dallas, TX, USA). NF-κB (1:1000; cat. no. ab16502) and TNFR2 (1:200; cat. no. ab8161) antibodies used for immunocytochemistry were purchased from Abcam (Cambridge, UK).

### Cell lines and evaluation of cytotoxicity by cell viability assay

Human ovarian adenocarcinoma cell line SKOV-3 was purchased from the American Type Culture Collection (ATCC, Rockville, MD, USA). The cells were cultivated in DMEM containing 10% (v/v) FBS and 1% (w/v) antibiotic-antimycotic in a 37 °C humidified CO2 incubator with 5% (v/v) CO2. Cell viability and the optimal dose (the half maximal inhibitory concentration, IC50) of CME for SKOV-3 cells were determined using the cell counting kit (CCK)-8 assay (Dojindo) as described previously [19]. SKOV-3 cells were grown for 24 h before the treatment of CME. The cells were seeded into 96-well plates at a density of 5 × 10^3^ cells/well in 0.1 mL media. After 24 h of incubation, the cells were exposure with various concentration of CME for 24 h. Treatment with 1% PBS was included as vehicle control. At the end of the treatment, 10 μL of CCK-8 solution was added to each well at the end of the treatment, and the plate was incubated for 2 h at 37 °C. The absorbance was measured at 450 nm wavelength using a Sunrise microplate absorbance reader (Tecan, Männedorf, Switzerland) relative to that of untreated control in triplicate experiments.

### Wound healing assay

Inhibition of migration activity in SKOV-3 treated with *C. militaris* extract was measured by wound-healing assay as described in our previous research [18]. SKOV-3 cells were seeded in a 24-well plate at a concentration of 2 × 10^4^ cells/well. When the cell destiny reached 90%, the surface of cell monolayer was scratched using micropipette tips to create linear gaps. After 2 washes with 2 mL of PBS were performed to flush out any suspended cells, SKOV-3 cells were treated with various concentrations of CME (125, 250, 500, 1000 μg/mL) for 24 h. The plates were imaged using the TissueFAXS system (TissueGnostics, Vienna, Austria). Wound closure was analyzed by measuring the healed area and the proportion of migrated cells using HistoQuest software (TissueGnostics).

### Apoptosis analysis by propidium iodide (PI)/Annexin V double staining

To determine the apoptotic induction by *C. militaris* in SKOV-3 cells, we used the Annexin V-fluorescein isothiocyanate (FITC) Apoptosis Detection Kit (Sigma, USA). Briefly, the cells were treated with *C. militaris* for 24 h, dissociated using trypsin, and washed twice with PBS. The cell suspension in PBS was centrifuged at 1500 rpm for 5 min, and the supernatant was carefully removed by pipetting. The cell pellet was resuspended in 500 μL Annexin-V binding buffer, and double-stained with 0.1 μg/mL Annexin V-FITC conjugate and 2 μg/mL PI for 10 min at 25 °C in the dark. Double-stained cell pellet 500 μL in tube loaded on Guava system (Millipore) for detecting the green/red fluorescence. The fluorescence of samples was immediately detected using Guava system (Millipore) at an excitation wavelength of 488 nm with a 530/30 nm band-pass filter to detect Annexin V and 670 nm high-pass filter to detect PI. We analyzed the rate of apoptosis using InCyte software of Guava system.

### Transmission electron microscopy (TEM)

SKOV-3 cells were seeded on a 100-mm culture dish (8 × 10^5^ cells/dish) and cultured in DMEM with 10% FBS and 1% antibiotic-antimycotic for 24 h. After SKOV-3 cells were grown, the cells were exposure with CME for 24 h. CME-treated SKOV-3 cells were sequentially fixed with 2.5% glutaraldehyde and 1% osmium tetroxide on ice for 2 h and washed with PBS. The fixed cells were then dehydrated in ethanol and propylene oxide series, embedded in an Epon 812 mixture, and polymerized in an oven at 70 °C for 24 h. The sections acquired from the polymerized blocks were collected on grids, counterstained with uranyl acetate and lead citrate, and examined with a Bio-HVEM system (JEM-1400Plus at 120 kV and JEM-1000BEF at 1000 kV, JEOL, JAPAN).

### Microarray analysis

Transcriptional profiling of the CME-treated ovarian cancer cells was carried out using a human twin 44 K cDNA chip as described in our previous study [21]. Total RNA was extracted from vehicle- or CME (500 μg/mL)-treated SKOV-3 ovarian cancer cells, and 50 mg RNA was subjected to cDNA synthesis in the presence of aminoallyl-dUTP by reverse transcription. The cDNA was coupled with Cy3 (vehicle) or Cy5 dye (CME-treated). The genes were thought to be differentially expressed when the global M and log2 (R/G) values exceeded |1.0| (two-fold) at *p* < 0.05. The Student’s *t*-test was applied to assess the statistical significance among the differentially expressed genes after CME treatment. To analyze the biological significance of these changes, the array data were categorized into specific gene groups.

### Gene ontology-based network analysis

To study the biological functions of the regulated genes through interaction network and The differentially expressed genes resulted via microarray in the SKOV-3 treated with CME or PBS we used the STRING database that can predict protein associations with direct binding or indirect interaction, such as participation in the same metabolic pathway or cellular process, through genomic context, high-throughput experiments, co-expression, and literature data (http://string.embl.de). Network generation was optimized based on the obtained expression profiles with an aim of producing highly connected networks.

### Immunoblotting

SKOV-3 cells treated with CME or PBS (vehicle) were lysed in ice-cold lysis buffer (20 mM Tris-HCl, pH 8.0) supplemented with 1% protease Inhibitor cocktail (sigma, P8340) and 1% phosphatase inhibitor cocktail 1,2 and 3 (sigma, P2850, P5726, and P0044) as described previously [38]. The cell homogenate was slowly inverted in 4 °C for 45 min before centrifugation (10 min, 12,000 rpm, 4 °C). And The expression of CME-induced apoptosis-related signaling proteins was examined using western blotting, as described previously [19]. The blotted membrane was blocked for 1 h with 5% (w/v) skimmed milk in TTBS (Tween-20 and Tris-buffered saline), followed by incubation with diluted primary antibodies, including anti- Bcl-xL (1:500), Bcl-2 (1:500), caspase-3 (1:500), caspase-9 (1:500), NF-κB (1:1000), TNFR1 (1:200), TNFR2 (1:200) and β-actin (1:1000), at room temperature for 2 h or at 4 °C overnight. The membrane was washed three times for 5 min each time with 0.1% (v/v) Tween-20 in TBS (TBST) before incubation with horseradish-peroxidase (HRP)-conjugated goat anti-mouse IgG or HRP-conjugated rabbit anti-goat IgG at a 1:2000 dilution in TBST containing 5% (w/v) skimmed milk at room temperature for 1 h. The membranes were rinsed three times with TBST for 5 min each, and an enhanced chemiluminescence system (Thermo Scientific, San Jose, CA, USA) was used to visualize the bands on a ChemiDoc MP system (Bio-Rad, Hercules, CA, USA). Densitometric analysis of the bands was performed using ImageJ software. Protein levels were quantitatively analyzed after normalization with β-actin level.

### Immunofluorescence microscopy

The cells were fixed with 4% formamide for 15 min at room temperature for 24 h after the establishment of an adherent culture. The cell membranes were permeabilized with 0.25% Triton X-100 in PBS for 10 min, blocked with TBST containing 1% bovine serum albumin (BSA; Sigma-Aldrich) for 30 min, and incubated with β-catenin primary antibody (Millipore, USA) for 1 h. The cells were incubated with Alexa Fluor 488-conjugated anti-mouse secondary antibody (Cell Signaling Technology) for 1 h in the dark. Following treatment with 4, 6-diamidino-2-phenylindole, fluorescence images were obtained under a confocal microscope (Nikon, Japan).

### Statistical analyses

GraphPad Prism (GraphPad, San Diego, CA, USA) was used for statistical analyses. Data were analyzed by one-way ANOVA followed by the Tukey-Kramer multiple comparisons test. The IC_50_ values were determined by nonlinear curve fitting using five data points and expressed as the mean ± standard deviation (SD).

## Results

### CME dose-dependently suppresses the growth of ovarian cancer cells

**Fig. 1:**
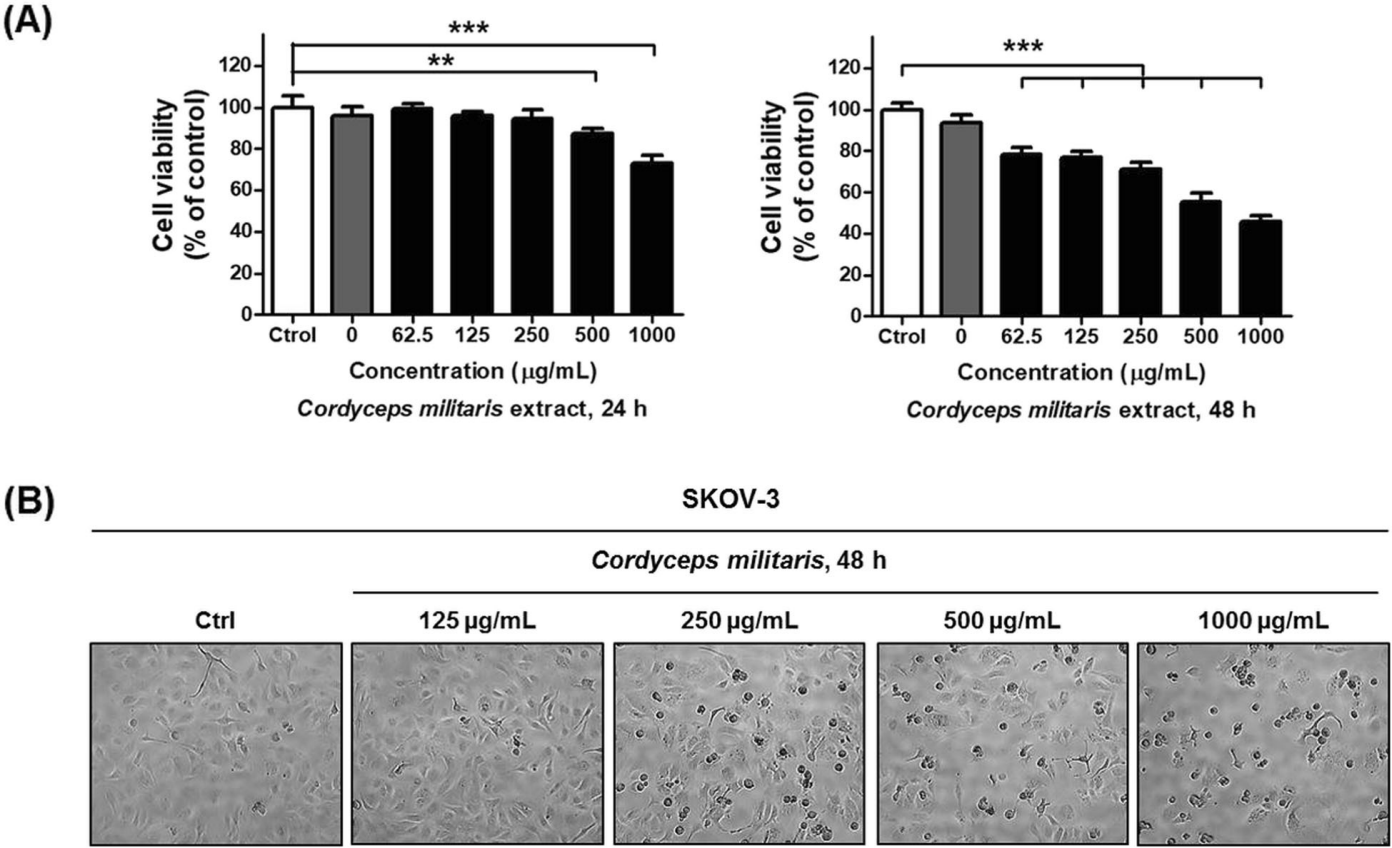
*Cordyceps militaris* extract dose-dependently inhibits viability and induces morphological changes in SKOV-3 cells (mixed type of HGSC, clear cell, and endometrioid). (**a**) SKOV-3 cells were exposed to 0 (vesicle), 62.5, 125, 250, 500, and 1000 μg/mL *C. militaris* extract for 24 and 48 h before estimation of cell number using the CCK-8 assay. The experiment was performed in triplicate. *C. militaris* significantly inhibited cell proliferation of SKOV-3 ovarian cancer cells. (**b**) Morphological changes of SKOV-3 were treated with *C. militaris* (125, 250, 500, and 1000 μg/mL) compared with control (vehicle). Magnification × 400. The statistics demonstrated that the percentage of the cells mainly represents treated cells, which was apparent when the percentage of control cells markedly decreased. Data are presented as means ± standard deviations from triplicate experiments. Statistical significance was considered that * means *p* < 0.05, ** means *p* < 0.01, *** *p* < 0.001, and ns means non-significance

To investigate the effects of CME on ovarian cancer cell proliferation, SKOV-3 cells were directly treated with 0, 62.5, 125, 250, 500, or 1000 μg/mL of CME for 24 and 48 h. As shown in Fig. 1a, CME inhibited the growth of cells in a dose-dependent manner within 48 h. Treatment with 1000 μg/mL extract for 48 h resulted in the inhibition of approximately half of SKOV-3 cell population. Thus, the half-maximal inhibitory concentration (IC_50_) was determined as 1000 μg/mL at 48 h (Fig. 1a). To observe the effects of CME on cancer cells, the morphologies of treated and untreated cells were compared under a light microscope. The morphology of SKOV-3 cells drastically changed after treatment with 250 μg/mL CME for 48 h (Fig. 1b). Multiple cells began to detach from the surface of the culture plate and appeared buoyant. Moreover, the cells were shrunken and showed reduced volume. These morphological changes preceded apoptosis. On the other hand, treatment with 125 μg/mL CME induced less drastic changes at 48 h.

### CME inhibits the migration activity of SKOV-3 ovarian cancer cells

**Fig. 2:**
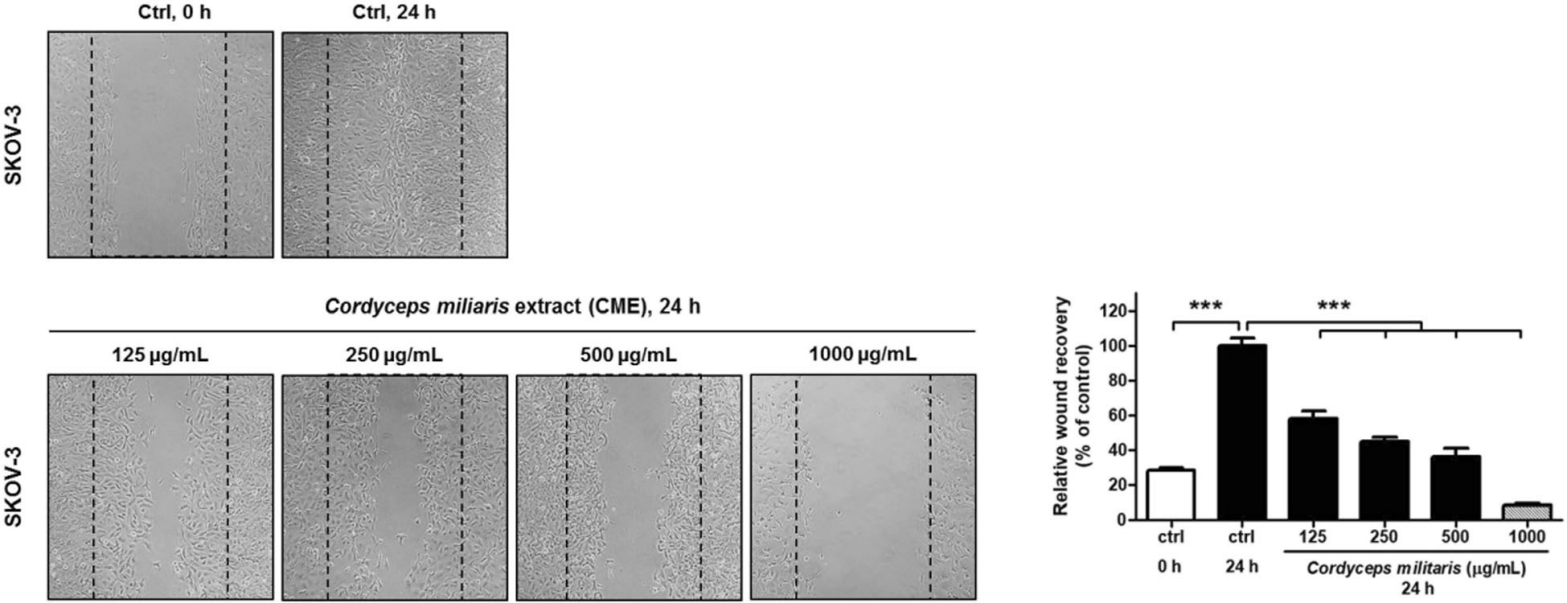
*Cordyceps militaris* extract inhibits the migration of SKOV-3 ovarian cancer cells in wound healing assay. To measure the migration activity, SKOV-3 cells were subjected to in vitro wound healing assay in the presence (125, 250, 500, 1000 μg/mL) or absence of CME for 24 h. Black-broken lines indicate initial wound edges. Migrating cells were photographed under an inverted microscope (magnification 100×). The statistics were shown the percentages of the cells represented by mainly treated cells which was apparent when the percentage of control cells markedly decreased. Data are presented as means ± standard deviations from triplicate experiments (*n* = 3). A value of ***p < 0.001 versus vehicle treatment was considered significant

We studied the effects of CME on the properties in SKOV-3 cells. To evaluate the potential biological relevance of the regulatory effect of CME on cellular migration, we performed wound healing assay with SKOV-3 cells after CME treatment. Results demonstrated that CME decreased the motility of SKOV-3 cells in a dose-dependent manner (Fig. 2). Thus, *C. militaris* may attenuate the mobility of human ovarian cancer cells.

### CME induces alteration in the expression of apoptotic genes in ovarian cancer cells

**Fig. 3:**
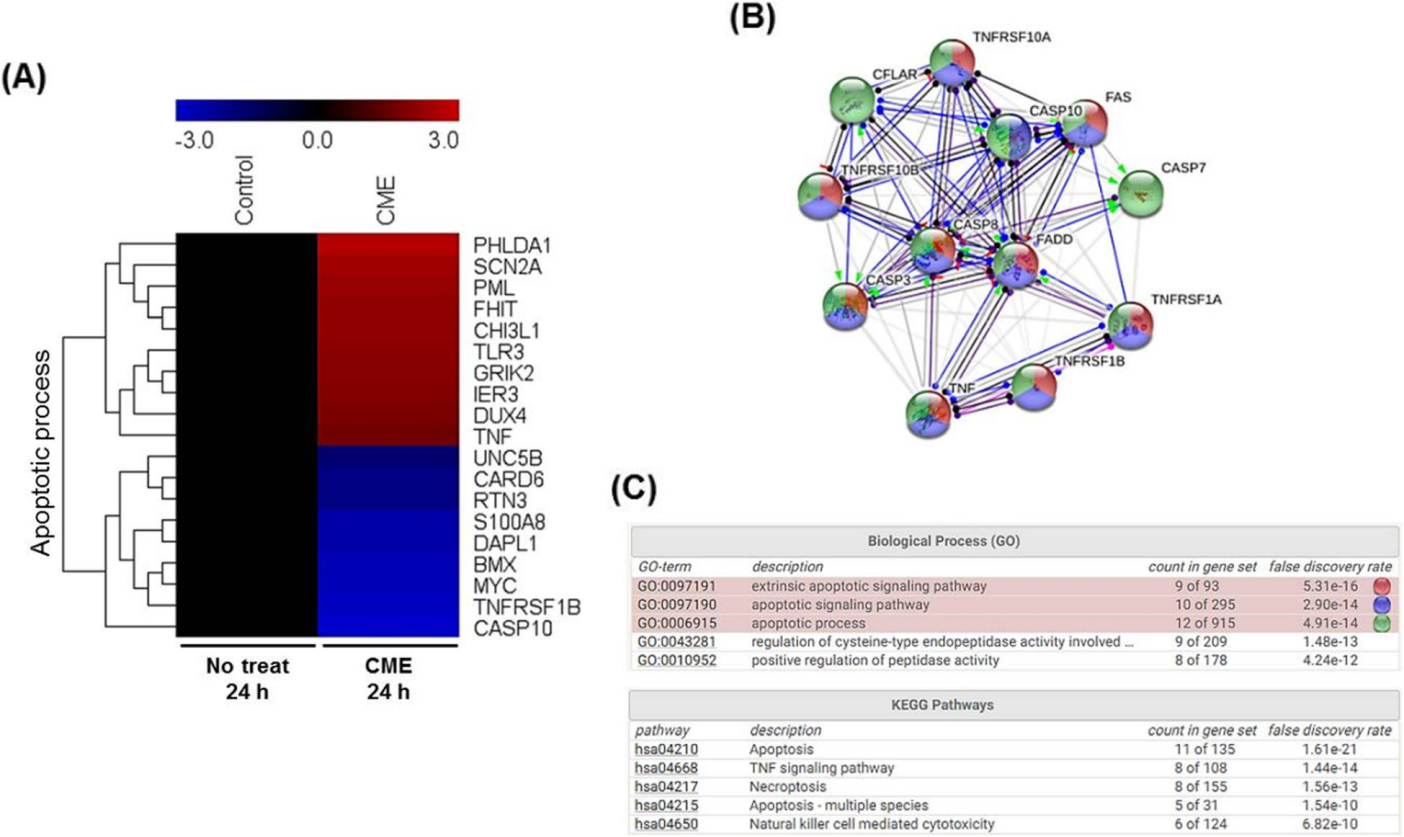
Microarray analysis to identify the alteration in the gene expression and signal network. **a** Hierarchical gene clustering was performed with the TM4 Microarray Software Suite (MeV) for CME-treated SKOV-3 cells. Heat map revealed the genes that underwent more than twofold changes after apoptosis in response to CME treatment. The red and blue colors represent more than threefold change in the expression of upregulated and downregulated genes, respectively. The ratios of gene profiles are presented as heat map (left panel) and gene expression pattern (right panel). **b** Combined screenshots from the STRING website, showing the results obtained after entering a set of 12 proteins thought to be involved in the apoptotic signaling pathway. The insets are shown as follows (from top to bottom): the accessory information available for a single protein, reported enrichment of functional connections among the set of proteins, and statistical enrichments detected in functional subsystems. **c** One enriched function was selected, and the corresponding protein nodes in the network were automatically highlighted

To investigate the expression of the genes affected by *C. militaris*, microarray analyses of CME (1000 μg/mL)-treated SKOV-3 ovarian cancer cells were conducted. Among the 58,201 genes assayed, 28,052 genes were expressed in the CME-treated cells. Among 7020 genes, *C. militaris* treatment upregulated and downregulated 1426 and 1372 genes, respectively, as compared with the observations reported in untreated control at 24 h. From the gene expression array data, we clustered the significantly affected core apoptosis-related genes (Fig. 3a). Genes that were upregulated or downregulated by more than twofold following CME treatment were categorized as “significant” in data mining. Biologically relevant features were constructed using the Excel-based differentially expressed gene analysis (ExDEGA) program. Lists of the threefold upregulated and downregulated apoptosis-related genes in CME-treated SKOV-3 ovarian cancer cells were uploaded to Multiple Experiment Viewer (MeV) tool for heat map and hierarchical cluster analyses (Fig. 3a). Heat maps and hierarchical clusters demonstrated 19 affected genes after CME treatment. Nine of these genes were found to be downregulated and 10 genes showed upregulated expression (Fig. 3a).

### Protein–protein interaction and gene ontology analysis in CME-treated ovarian cancer cells

Analyses of heat maps and hierarchical clusters revealed the correlation between TNF-α and TNFRSF1B expression in CME-treated SKOV-3 cells (Fig. 3a). Based on these results, protein-protein interaction and gene ontology analysis were performed using the STRING database. Twelve genes. Including those encoding TNF-α and TNFRSF1B, were found to interact with each other (Fig. 3b). Pathway analysis comparing non-treated and CME-treated SKOV-3 cells revealed the involvement of all related proteins in the regulation of apoptotic process (GO: 0006915; false discovery rate *P* = 4.91^− 14^: CASP3, CASP7, CASP8, CASP10, CFLAR, FADD, FAS, TNF, TNFRSF1A, TNFRSF1B, TNFRSF10A, TNFRSF10B), especially extrinsic apoptotic signaling pathway (GO: 0097191; false discovery rate *P* = 5.31^− 14^: CASP3, CASP8, FADD, FAS, TNF, TNFRSF1A, TNFRSF1B, TNFRSF10A, TNFRSF10B) (Fig. 3c). In the Kyoto Encyclopedia of gene and genome (KEGG) analysis, related genes were involved in apoptosis, TNF-α signaling pathway, necroptosis, and natural killer cell-mediated cytotoxicity (Fig. 3c). Thus, we hypothesized that *C. militaris* induced TNF-α/TNFR signal transduction pathway-mediated apoptosis of ovarian cancer cells.

### CME induces apoptosis of ovarian cancer cells

**Fig. 4:**
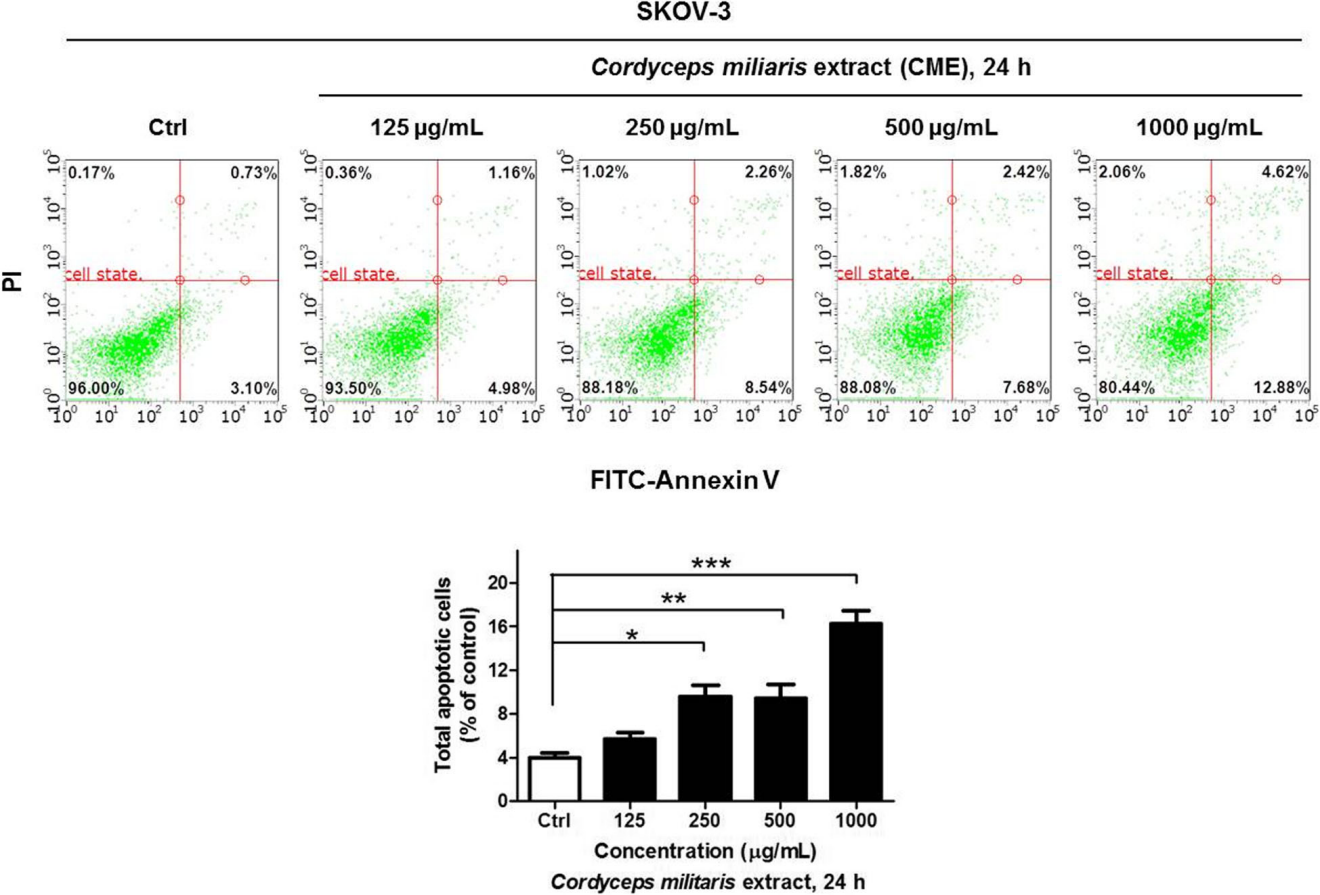
*Cordyceps militaris* extract induces apoptosis of SKOV-3 ovarian cancer cells. Flow cytometry analysis was performed with SKOV-3 cells after treatment with the indicated concentrations of CME for 24 h. The cells were stained using Annexin V-FITC Apoptosis Detection Kit and the apoptosis array was determined by Guava system (Millipore). The results are expressed as the percentage of early and late apoptotic cells, which increased as the percentage of live cells markedly decreased. Data are presented as means ± standard deviations from triplicate experiments (*n* = 3) and were normalized to controls and represented as the mean ± SD for three independent experiments (*p < 0.05, **p < 0.01, ***p < 0.001)

The apoptotic effect of *C. militaris* on SKOV-3 ovarian cancer cells was analyzed with Annexin V and PI staining and flow cytometry after 24 h treatment with 0 (control), 125, 250, 500, and 1000 μg/mL of CME. The relative proportion of non-viable cells was quantitatively measured as the cells at the early stage of apoptosis (Annexin V-stained, non-disrupted cells) or those entering the late stage of apoptosis (disrupted or lysed cells). The treatment of cells with 125 μg/mL CME for 24 h resulted in no drastic change in Annexin V-stained viable fraction (96 to 93%) (Fig. 4). However, the cells treated with 250, 500, 1000 μg/mL CME for 24 h showed a marked shift from the normal state to apoptotic stage (3.8 to 10.8%, 10.1, and 17.5%). The viable fraction in the cells treated with 1000 μg/mL CME reduced from 96 to 80.44%. Thus, 250, 500, 1000 μg/mL CME treatment for 24 h induced the apoptosis of ovarian cancer cells (Fig. 4).

### CME-treated ovarian cancers reveal apoptotic bodies under TEM

**Fig. 5:**
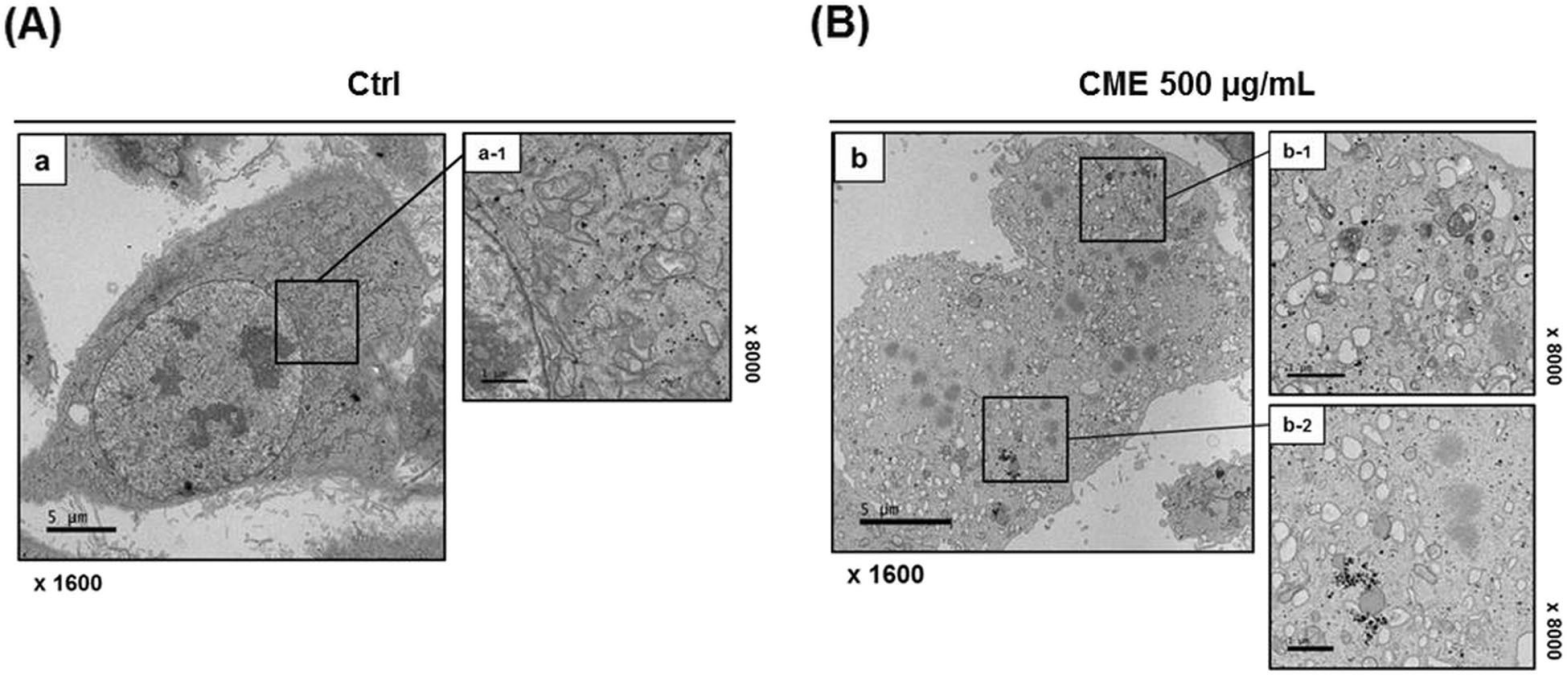
Morphological changes in the ultrastructural appearance of apoptotic bodies in CME-treated ovarian cancer cells with transmission electron microscopy (TEM). **a** Untreated SKOV-3 and (**b**) CME-treated SKOV-3 cells (500 μg/mL CME concentration) were analyzed by TEM after 24 h. The typical apoptotic bodies in CME-treated cells were observed as spherical protuberances containing fragmented and segregated chromatin clumps separated from cell surface. Mitochondrial disruption and autophagosomes were observed in CME-treated SKOV-3 cells. Representative images are shown

To confirm the development of apoptotic bodies after *cordyceps militaris* treatment and to visualize the ultrastructural changes in SKOV-3 apoptotic cells, we used TEM (Fig. 5). Apoptotic bodies were observed in cells treated with 500 μg/mL CME; these bodies were spherical protuberances containing fragmented and segregated chromatin clumps, which separated from the cell surface (Fig. 5a). CME*-*exposed cells showed damaged mitochondria and autophagosomes containing dense organelles a day after treatment (Fig. 5a). Conversely, the untreated SKOV-3 cells had intact plasma membranes and ordered chromatin (Fig. 5b).

### CME increases the expression of apoptosis-related proteins in ovarian cancer cells

**Fig. 6:**
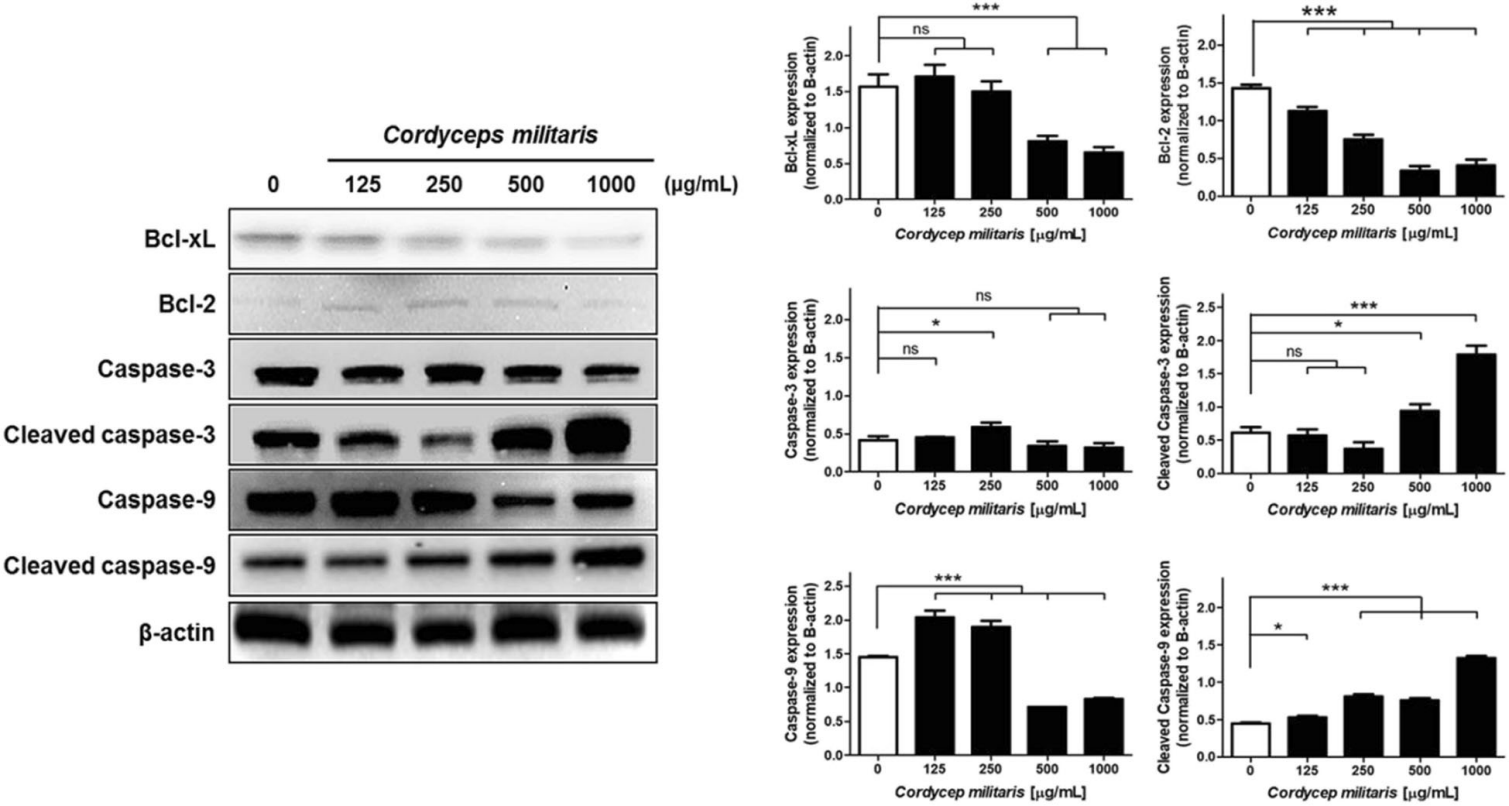
*Cordyceps militaris* extract induces alteration in the expression of apoptotic proteins. The indicated cell lines were exposed to 0, 125, 250, 500, and 1000 μg/mL *Cordyceps militaris* extract for 12 h, and the whole-cell protein lysates were harvested and used for western blot analysis for Bcl-xL, Bcl-2, cleaved caspase-9, and caspase-3. Data are presented as means ± standard deviations from triplicate experiments (n = 3) and were normalized to controls and represented as the mean ± SD for three independent experiments (*p < 0.05, **p < 0.01, ***p < 0.001)

To study the mechanism underlying the effects of *C. militaris* on cell proliferation and apoptosis induction, SKOV-3 ovarian cancer cells were treated with different doses of CME (0, 125, 250, 500, and 1000 μg/mL), followed by analysis of protein expression. The levels of Bcl-xL, Bcl-2, and Bax pro-apoptotic members were analyzed with immunoblotting. As a result, we found that the expression levels of cleaved caspase-3 and caspase-9 significantly increased after treatment with CME (Fig. 6). Taken together, these results imply that *C. militaris* induced apoptosis of cells through the Bcl-xL, Bcl-2 and caspase-dependent pathways (Fig. 6). TNF-α and TNFR have been shown to be key positive regulators of the extrinsic apoptosis pathway through the inhibition of the activation of caspases in human ovarian cancer cells. Therefore, we evaluated whether *C. militaris* influences the levels of TNFR in ovarian cancer cells.

### Upregulation of TNFR1 expression by CME induces apoptosis of cells through the suppression of the nuclear translocation of NF-κB

**Fig. 7:**
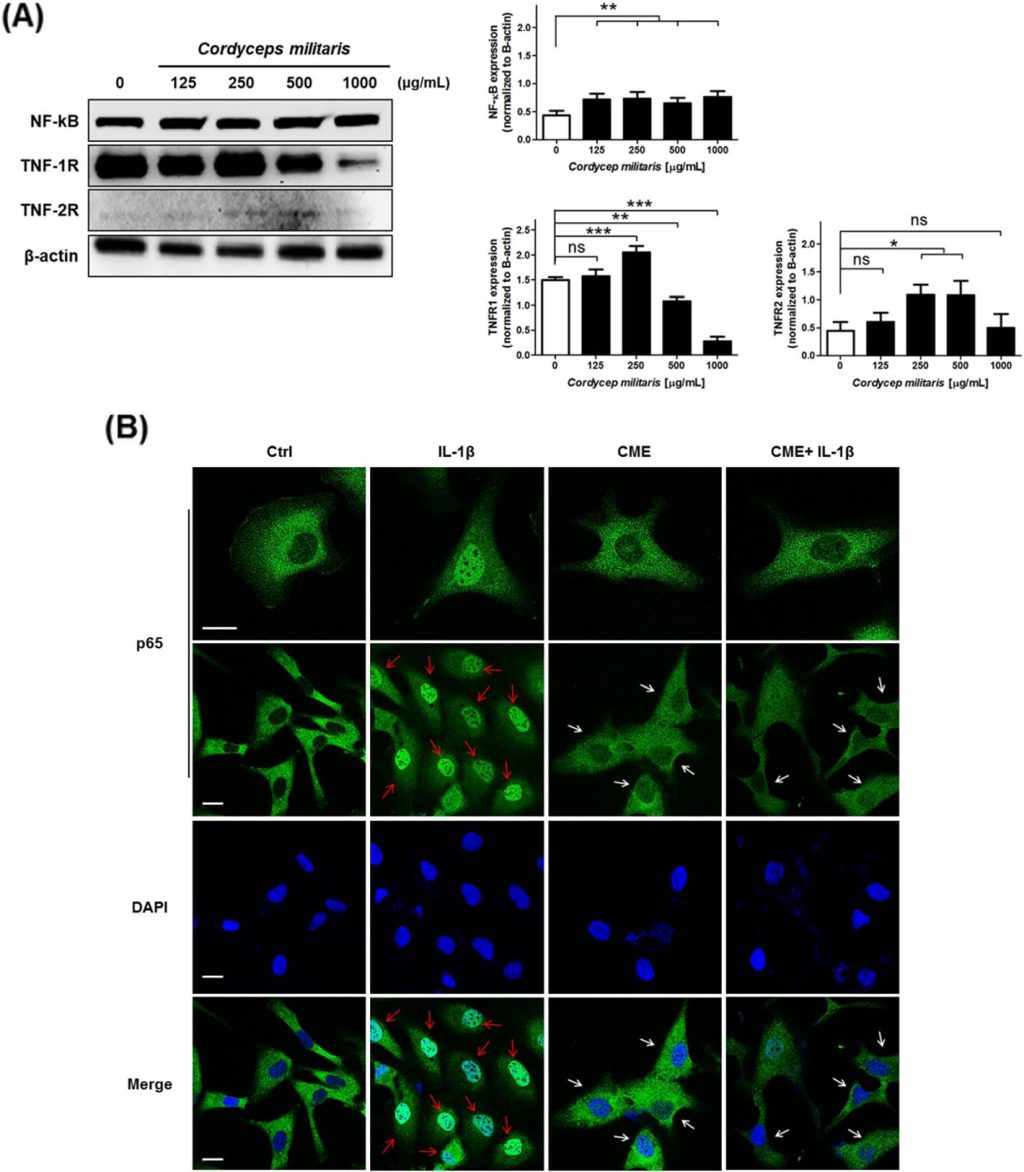
*Cordyceps miliaris* extract suppresses the nuclear translocation of NF-kB (p65) via upregulation of TNF1R expression. **a** Effect of TNF1R on the nuclear translocation of NF-kB (p65) in CME-treated SKVO-3 cells. **b** Immunofluorescence micrographs of NF-κB (p65) translocation in SKOV-3 cells. NF-κB (p65) translocation into the nucleus was detected with an anti-NF-κB (p65) antibody (green fluorescence); the inhibition of this signaling pathway is shown by white arrows, whereas activation is indicated with red arrows. Scale bar, 10 μm. Data are presented as means ± standard deviations from triplicate experiments (*n* = 3). The data represent the mean ± SD from three independent experiments (*p < 0.05, **p < 0.01, and ***p < 0.001)

To investigate whether the TNF-α/TNFR/NF-κB axis is functionally linked to caspase signaling, we examined the effect TNF-α/TNFR on NF-κB activation. SKOV-3 ovarian cancer cells are primary ascitic ovarian tumor cells that express TNFR1 but not TNFR2 [39]. Therefore, we confirmed the alternation in TNFR1 and TNFR2 expression after CME treatment of SKOV-3 cells. In addition, the activity of NF-κB was determined by the binding of TNF-α to TNFR1 or TNFR2. We also identified the changes in the levels of NF-κB expression. TNFR1 expression, upregulated by 250 μg/mL CME treatment, induced both membrane disruption via apoptosis activation and downregulation TNFR1 expression (Fig. 7a). However, the extrinsic apoptotic signaling pathway was upregulated in a dose-dependent manner (Fig. 6). Next, we investigated the effects of TNFR1 on NF-κB signaling after CME treatment of ovarian cancer cells. The changes in NF-κB expression in CME-treated SKOV-3 cells were examined. As a result, we found that the protein expression of NF-κB was not significantly changed (Fig. 7a). To confirm the activation of NF-kB, we detected its translocation to the nucleus. As the expression of TNFR2 was absent in SKOV-3 cells, we treated cells with IL-1 to induce translocation of NF-kB. However, the treatment of SKOV-3 cells with CME decreased the nuclear translocation of NF-κB (Fig. 7b). These results indicate that *C. militaris* attenuated the TNF-α/TNFR1-mediated NF-κB phosphorylation to induce extrinsic apoptosis pathway in SKOV-3 ovarian cancer cells.

## Discussion

Epithelial ovarian cancer (EOC) is a malignant gynecological tumor. Although the global incidence rate of EOC ranks third, its mortality rate ranks first among female genital malignancies [40]. EOC is mainly treated by surgery combined with chemotherapy, radiotherapy, and some immune modulators [41]. However, intrinsic or acquired resistance to chemotherapy often critically limits the efficacy and outcome of treatment [42]. Also, almost all chemotherapeutic agents used in the treatment of ovarian cancer develop resistance mechanisms that are responsible for recurrence. The mechanisms of cellular resistance include reduced intracellular accumulation of the drug, increased DNA repair and altered oncogene and regulatory protein expression [43]. In addition, anti-apoptotic genes expression is downregulated and mutations in the apoptotic pathway is increased contributing to impaired DNA damage detection and apoptosis induction [44]. Recently, Nuclear Factor-kappa B (NF-κB) has been identified as a key player in resistance mechanisms [45]. NF-κB activity inversely correlated with cellular sensitivity to chemotherapy in carcinoma cell lines [46].

NF-κB has also oncogenic function, which has been well documented in many cancers, and affects on activating multiple target genes involved in anti-apoptosis, cell-cycle progression, and angiogenesis [47]. In ovarian cancer, the increased activity of NF-κB has been reported to be a predictor of poor disease progression and to confer resistance to cisplatin-induced apoptosis [48]. Therefore, cisplatin enhanced the DNA binding activity of NF-κB, through increased expression and activation of the protein, thereby limiting its own potential efficacy. Treatment to various cancer cells with AKT-NF-κB inhibitor abrogated the increased NF-κB activity, sensitizing them to cisplatin induced apoptosis [21, 49]. It suggests that inhibition of NF-κB upregulates pro-apoptotic protein activation in ovarian cancer, supporting the sensitivity of chemotherapy for ovarian cancer.

*C. militaris* has been reported to exhibit various biological properties, including antitumor, antiviral, antioxidant, and anti-inflammatory activities [50]. A few studies have reported the anticancer activity of *C. militaris* [9, 15, 51]. In the present study, we investigated the anticancer effect of *C. militaris* on SKOV-3 human ovarian cancer cells. We found that *C. militaris* reduced the viability and migration of human ovarian cancer cells in a dose- and time-dependent manner, indicative of its potential ability to mediate apoptosis (Figs. 1, 2). In addition, flow cytometry analysis revealed that approximately 20% SKOV-3 and OVCAR-3 cells exhibited early- and late-phase apoptosis after the exposure to *C. militaris* (1000 μg/mL) for 24 h (Fig. 4). We used TEM to visualize the apoptotic bodies in CME-treated SKOV-3 cells (Fig. 5). The untreated control cells showed normal organelles without apoptotic bodies.

Several studies have shown that cordycepin, a major active component in *C. militaris*, represses the expression of inflammation-related genes through the suppression of NF-kB activation [11]. These data suggest that *C. militaris* is involved in the regulation of NF-κB signaling pathway. However, the effect of *C. militaris* on the key players of NF-κB signaling pathway remains unknown, and further researches are warranted to evaluate the detailed underlying molecular mechanism. Although *C. militaris* -induced cell death has been previously reported, the underlying molecular mechanism has not been elucidated in ovarian cancer. Here, we tried to understand the fundamental mechanism underlying the apoptotic effects of *C. militaris* and examined the relationship between TNFR1 expression and NF-κB activation.

The binding of TNF-α to TNFR results in the dissociation of IκB from NF-κB and the activation of NF-κB, which is translocated to the nucleus [52]. However, TNF-α functions as a trimer, and binds to one of its two receptors TNFR1 or TNFR2 [53]. In addition, TNF-α performs several biological functions depending on its binding to receptor subtypes [54, 55]. Our results showed that *C. militaris* upregulated the expression of TNFR1 (Fig. 7a), and reduced NF-κB translocation into the nucleus induced after IL-1β treatment (Fig. 7b). As a result, we observed an increase in the level of cleaved caspase-3 and cleaved caspase-9 and a decrease in Bcl-xL and Bcl-2 levels (Fig. 6). These findings indicate that *C. militaris* suppressed NF-κB signaling pathway by downregulating the interaction between TNF-α and TNFR1 in SKOV-3 ovarian cancer cells.

## Conclusion

In summary, our results demonstrate that *C. militaris* triggers TNF-α/TNFR1-mediated apoptosis by suppressing the NF-κB signaling pathway and promotes the cleavage of caspase-3 and caspase-9 through the induction of the extrinsic apoptotic pathway in ovarian cancer cells. Our findings describe the molecular mechanisms underlying the apoptosis induced by *C. militaris*, and may provide a theoretical basis for the application of *C. militaris* derivatives for cancer treatment.

## Data Availability

The datasets used and/or analyzed during the current study available from the corresponding author on reasonable request.

## Abbreviations

Bcl: B cell lymphoma
CME: *Cordyceps militaris* extract
IL: Interleukin
NF-κB: Nuclear factor kappa B
TNF: Tumor necrosis factor
TNFR: Tumor necrosis factor receptor
